# *N*-Carbethoxy-*N*′-(3-phenyl-1*H*-1,2,4-triazol-5-yl)thiourea

**DOI:** 10.1107/S1600536810002369

**Published:** 2010-01-23

**Authors:** Anton V. Dolzhenko, Geok Kheng Tan, Lip Lin Koh, Anna V. Dolzhenko, Wai Keung Chui

**Affiliations:** a Department of Pharmacy, Faculty of Science, National University of Singapore, 18 Science Drive 4, Singapore 117543, Singapore; b Department of Chemistry, Faculty of Science, National University of Singapore, 3 Science Drive 3, Singapore 117543, Singapore

## Abstract

The title compound {systematic name: ethyl *N*-[*N*-(3-phenyl-1*H*-1,2,4-triazol-5-yl)carbamothio­yl]carbamate}, C_12_H_13_N_5_O_2_S, exists in the 3-phenyl-5-thio­ureido-1*H*-1,2,4-triazole tautomeric form stabilized by intra­molecular hydrogen bonding between the endocyclic NH H atom and the thio­ureido S atom. The mol­ecular structure is also stabilized by intra­molecular N—H⋯O=C hydrogen bonds arranged in an *S*(6) graph-set motif within the carbethoxy­thio­urea moiety. The mean planes of the phenyl and 1,2,4-triazole rings make a dihedral angle of 7.61 (11)°. In the crystal, the mol­ecules form two types of inversion dimers. Inter­molecular hydrogen bonds are arranged in *R*_2_^2^(6) and *R*_2_^2^(8) graph-set motifs, together forming a network parallel to (111).

## Related literature

For the synthesis, tautomerism and crystal structure studies of related 1,2,4-triazoles, see: Dolzhenko *et al.* (2007 ▶, 2009**a* ▶,*b* ▶,c* ▶). For the structures of related carbethoxy­thio­ureas, see: Huang *et al.* (2009 ▶); Lin *et al.* (2004 ▶, 2007 ▶); Su *et al.* (2006 ▶); Zhang *et al.* (2003 ▶, 2007 ▶). For the graph-set analysis of hydrogen bonding, see: Bernstein *et al.* (1995 ▶).
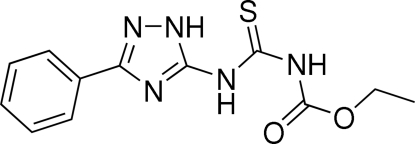


## Experimental

### 

#### Crystal data

C_12_H_13_N_5_O_2_S
*M* *_r_* = 291.33
Triclinic, 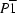

*a* = 5.9929 (3) Å
*b* = 9.4200 (5) Å
*c* = 12.2000 (7) Å
α = 91.818 (1)°
β = 92.585 (1)°
γ = 101.083 (1)°
*V* = 674.62 (6) Å^3^
*Z* = 2
Mo *K*α radiation
μ = 0.25 mm^−1^
*T* = 100 K
0.56 × 0.24 × 0.12 mm

#### Data collection

Bruker SMART APEX CCD diffractometer
Absorption correction: multi-scan (*SADABS*; Sheldrick, 2001 ▶) *T*_min_ = 0.873, *T*_max_ = 0.971
8943 measured reflections
3092 independent reflections
2828 reflections with *I* > 2σ(*I*)
*R*_int_ = 0.028

#### Refinement

*R*[*F* ^2^ > 2σ(*F* ^2^)] = 0.040
*wR*(*F* ^2^) = 0.102
*S* = 1.06
3092 reflections
194 parameters
H atoms treated by a mixture of independent and constrained refinement
Δρ_max_ = 0.59 e Å^−3^
Δρ_min_ = −0.20 e Å^−3^

### 

Data collection: *SMART* (Bruker, 2001 ▶); cell refinement: *SAINT* (Bruker, 2001 ▶); data reduction: *SAINT*; program(s) used to solve structure: *SHELXS97* (Sheldrick, 2008 ▶); program(s) used to refine structure: *SHELXL97* (Sheldrick, 2008 ▶); molecular graphics: *SHELXTL* (Sheldrick, 2008 ▶); software used to prepare material for publication: *SHELXTL*.

## Supplementary Material

Crystal structure: contains datablocks I, global. DOI: 10.1107/S1600536810002369/gw2076sup1.cif

Structure factors: contains datablocks I. DOI: 10.1107/S1600536810002369/gw2076Isup2.hkl

Additional supplementary materials:  crystallographic information; 3D view; checkCIF report

## Figures and Tables

**Table 1:** Hydrogen-bond geometry (Å, °)

*D*—H⋯*A*	*D*—H	H⋯*A*	*D*⋯*A*	*D*—H⋯*A*
N5—H5*N*⋯S1^i^	0.81 (2)	2.58 (2)	3.3739 (13)	166.0 (17)
N4—H4*N*⋯O2	0.84 (2)	1.97 (2)	2.6448 (16)	137.3 (18)
N3—H3*N*⋯S1	0.84 (2)	2.67 (2)	3.0926 (13)	113.0 (16)
N3—H3*N*⋯N2^ii^	0.84 (2)	2.32 (2)	2.9838 (18)	136.5 (18)

Symmetry codes: (i) 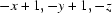; (ii) 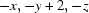.

**Fig. 1.**
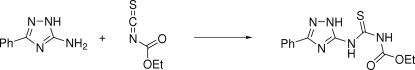
Synthesis of N-carbethoxy-N'-(3-phenyl-1H-1,2,4-triazol-5-yl)thiourea.

**Fig. 2.**
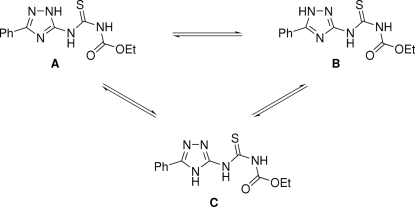
Annular tautomerism in N-carbethoxy-N'-(3(5)-phenyl-1(4)H-1,2,4-triazol-5(3)-yl)thiourea.

**Fig. 3.**
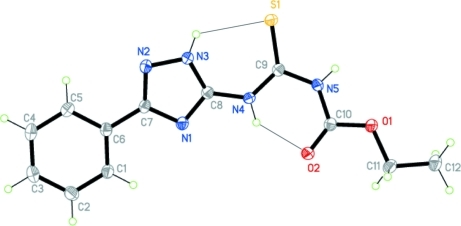
The molecular structure of N-carbethoxy-N'-(3-phenyl-1H-1,2,4-triazol-5-yl)thiourea, showing the atomic numbering scheme. Displacement ellipsoids are drawn at the 50% probability level.

**Fig. 4.**
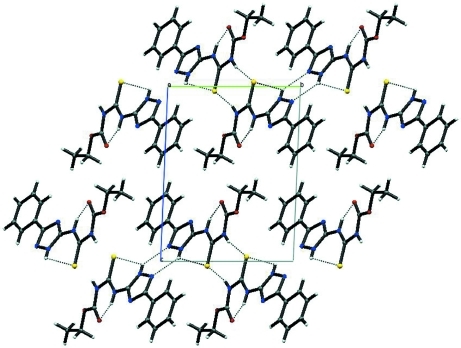
Crystal packing in the cell (view along axis a).

## supplementary crystallographic information

### Comment

Annular tautomerism of 1,2,4-triazoles in solutions (Dolzhenko et al., 2009a) and crystalline state (Dolzhenko et al., 2009b,c) is a subject of our continuous investigations. Recently, we reported the crystal structure of 3(5)-amino-5(3)-phenyl-1H-1,2,4-triazole (Dolzhenko et al., 2009b). Both 3-amino-5-phenyl- and 5-amino-3-phenyl-1H-1,2,4-triazole tautomeric forms were found to coexist in the crystal. Herein we study the related structure with carbethoxythiourea moiety presented instead of the amino group. Due to annular tautomerism, there is theoretical possibility for existence of three tautomeric forms viz. N-carbethoxy-N'-(3-phenyl-1H-1,2,4-triazol-5-yl)thiourea (A), N-carbethoxy-N'-(5-phenyl-1H-1,2,4-triazol-3-yl)thiourea (B) and N-carbethoxy-N'-(3-phenyl-4H-1,2,4-triazol-5-yl)thiourea (C) (Figure 2). Unlike 3(5)-amino-5(3)-phenyl-1H-1,2,4-triazole, only one tautomeric form A was identified in the crystal (Figure 3). The N3—H···S1 hydrogen bonds between the endocyclic N(3)H proton of the triazole ring and the thioureido sulfur S1 atom (Figure 3 and 4, Table 1) are arranged in a S(6) graph-set motif (Bernstein et al., 1995) stabilizing this tautomer. Interestingly, structurally similar carbethoxythioureido substituted pyrazole (Huang et al., 2009) does not possess this motif and crystallizes as a tautomer with the carbethoxythiourea moiety at position 5 of the ring.

The triazole ring is essentially planar with an r.m.s. deviation of 0.0058 Å. Its mean plane makes a dihedral angle of 7.61 (11)° with the phenyl ring.

The C—N bonds of the thiourea group have unequal lengths: the C9—N4 bond is significantly shorter (1.3440 (18) Å) compare to the C9—N5 bond (1.3811 (18) Å). The configuration of the carbethoxythiourea group of the title compound is similar to those reported for the similar structures (Huang et al., 2009; Lin et al., 2007; Lin et al., 2004; Su et al., 2006; Zhang et al., 2007; Zhang et al., 2003). The triazole ring and the thiocarbonyl lie in (Z)-configuration across the thiourea C9—N4 bond; while the carbethoxy and thiocarbonyl groups adopt (E)-configuration across the C9—N5 bond. This configuration is stabilized by an intramolecular N4—H···O2═C10 hydrogen bond (Figure 3 and 4, Table 1) making a S(6)graph-set motif, which is common for carbethoxythioureas (Huang et al., 2009; Lin et al., 2007; Lin et al., 2004; Su et al., 2006; Zhang et al., 2007; Zhang et al., 2003).

In the crystal, the molecules form two types of cyclic dimmers (Figure 4, Table 1). The N2—N3H sides of two molecules are connected by intermolecular hydrogen bonds making the R_2_^2^(6) graph-set motif. Atom N5 is also involved in intermolecular N—H···S interactions with the thiocarbonyl atom S1 of adjacent molecule making another pair with the R_2_^2^(8) graph-set motif similar to those observed in other carbethoxythioureas (Huang et al., 2009; Lin et al., 2007; Lin et al., 2004; Su et al., 2006; Zhang et al., 2007; Zhang et al., 2003). Together, these hydrogen bonds connect molecules in a network parallel to the (111) plane.

### Experimental

The title compound was synthesized by nucleophilic addition of 3(5)-amino-5(3)-phenyl-1*H*-1,2,4-triazole (Dolzhenko *et al.*, 2007) to ethoxycarbonyl isothiocyanate in DMF solution at room temperature (Figure 1). Single crystals suitable for crystallographic analysis were grown by recrystallization from toluene.

### Refinement

All the H atoms attached to the carbon atoms were constrained in a riding motion approximation [0.95 Å for C_aryl_—H, 0.99 Å for methylenic protons and 0.98 Å for methyl group; *U*_iso_(H) = 1.2*U*_eq_(C_aryl_), *U*_iso_(H) = 1.2*U*_eq_(C_methylenic_) and *U*_iso_(H) = 1.5*U*_eq_(C_methyl_)] while the N-bound H atoms were located in a difference map and refined freely.

### Crystal data

**Table:**

C_12_H_13_N_5_O_2_S	*Z* = 2
*M**_r_* = 291.33	*F*(000) = 304
Triclinic, *P*1	*D*_x_ = 1.434 Mg m^−^^3^
Hall symbol: -P 1	Melting point: 454 K
*a* = 5.9929 (3) Å	Mo *K*α radiation, λ = 0.71073 Å
*b* = 9.4200 (5) Å	Cell parameters from 4121 reflections
*c* = 12.2000 (7) Å	θ = 2.7–27.5°
α = 91.818 (1)°	µ = 0.25 mm^−^^1^
β = 92.585 (1)°	*T* = 100 K
γ = 101.083 (1)°	Rod, colourless
*V* = 674.62 (6) Å^3^	0.56 × 0.24 × 0.12 mm

### Data collection

**Table:**

Bruker SMART APEX CCD diffractometer	3092 independent reflections
Radiation source: fine-focus sealed tube	2828 reflections with *I* > 2σ(*I*)
graphite	*R*_int_ = 0.028
φ and ω scans	θ_max_ = 27.5°, θ_min_ = 1.7°
Absorption correction: multi-scan (*SADABS*; Sheldrick, 2001)	*h* = −7→7
*T*_min_ = 0.873, *T*_max_ = 0.971	*k* = −12→12
8943 measured reflections	*l* = −15→15

### Refinement

**Table:**

Refinement on *F*^2^	Primary atom site location: structure-invariant direct methods
Least-squares matrix: full	Secondary atom site location: difference Fourier map
*R*[*F*^2^ > 2σ(*F*^2^)] = 0.040	Hydrogen site location: inferred from neighbouring sites
*wR*(*F*^2^) = 0.102	H atoms treated by a mixture of independent and constrained refinement
*S* = 1.06	*w* = 1/[σ^2^(*F*_o_^2^) + (0.0557*P*)^2^ + 0.2404*P*] where *P* = (*F*_o_^2^ + 2*F*_c_^2^)/3
3092 reflections	(Δ/σ)_max_ = 0.001
194 parameters	Δρ_max_ = 0.59 e Å^−^^3^
0 restraints	Δρ_min_ = −0.20 e Å^−^^3^

### Special details

**Table:**

Geometry. All e.s.d.'s (except the e.s.d. in the dihedral angle between two l.s. planes) are estimated using the full covariance matrix. The cell e.s.d.'s are taken into account individually in the estimation of e.s.d.'s in distances, angles and torsion angles; correlations between e.s.d.'s in cell parameters are only used when they are defined by crystal symmetry. An approximate (isotropic) treatment of cell e.s.d.'s is used for estimating e.s.d.'s involving l.s. planes.
Refinement. Refinement of *F*^2^ against ALL reflections. The weighted *R*-factor *wR* and goodness of fit *S* are based on *F*^2^, conventional *R*-factors *R* are based on *F*, with *F* set to zero for negative *F*^2^. The threshold expression of *F*^2^ > 2σ(*F*^2^) is used only for calculating *R*-factors(gt) *etc*. and is not relevant to the choice of reflections for refinement. *R*-factors based on *F*^2^ are statistically about twice as large as those based on *F*, and *R*- factors based on ALL data will be even larger.

### Fractional atomic coordinates and isotropic or equivalent isotropic displacement parameters (Å^2^)

**Table:**

	*x*	*y*	*z*	*U*_iso_*/*U*_eq_	
S1	0.26167 (6)	0.64734 (4)	−0.02591 (3)	0.02072 (12)	
O1	0.58945 (18)	0.44334 (11)	0.28112 (8)	0.0204 (2)	
O2	0.31338 (18)	0.55681 (11)	0.33852 (8)	0.0217 (2)	
N1	−0.1894 (2)	0.78466 (13)	0.22558 (10)	0.0180 (3)	
N2	−0.1881 (2)	0.93860 (13)	0.08657 (10)	0.0196 (3)	
N3	−0.0355 (2)	0.84999 (13)	0.07092 (10)	0.0190 (3)	
H3N	0.052 (3)	0.866 (2)	0.0191 (17)	0.033 (5)*	
N4	0.1037 (2)	0.66518 (13)	0.17503 (10)	0.0179 (3)	
H4N	0.111 (3)	0.643 (2)	0.2411 (17)	0.028 (5)*	
N5	0.3803 (2)	0.52952 (13)	0.15529 (10)	0.0187 (3)	
H5N	0.458 (3)	0.494 (2)	0.1140 (15)	0.020 (4)*	
C1	−0.5206 (3)	0.91936 (16)	0.33573 (12)	0.0208 (3)	
H1	−0.4508	0.8519	0.3742	0.025*	
C2	−0.6883 (3)	0.97955 (17)	0.38396 (13)	0.0246 (3)	
H2	−0.7334	0.9528	0.4553	0.030*	
C3	−0.7902 (3)	1.07873 (17)	0.32819 (14)	0.0254 (3)	
H3	−0.9047	1.1199	0.3614	0.030*	
C4	−0.7245 (3)	1.11747 (16)	0.22403 (13)	0.0237 (3)	
H4	−0.7936	1.1857	0.1861	0.028*	
C5	−0.5586 (3)	1.05716 (16)	0.17507 (12)	0.0204 (3)	
H5	−0.5154	1.0833	0.1034	0.024*	
C6	−0.4547 (2)	0.95789 (15)	0.23090 (12)	0.0175 (3)	
C7	−0.2773 (2)	0.89424 (15)	0.17999 (12)	0.0172 (3)	
C8	−0.0381 (2)	0.76241 (15)	0.15491 (11)	0.0173 (3)	
C9	0.2436 (2)	0.61501 (15)	0.10695 (11)	0.0170 (3)	
C10	0.4197 (2)	0.51397 (15)	0.26620 (12)	0.0181 (3)	
C11	0.6496 (3)	0.42025 (17)	0.39532 (12)	0.0246 (3)	
H11A	0.5123	0.3763	0.4334	0.030*	
H11B	0.7187	0.5134	0.4339	0.030*	
C12	0.8169 (3)	0.32036 (17)	0.39450 (14)	0.0250 (3)	
H12A	0.7455	0.2281	0.3572	0.037*	
H12B	0.8636	0.3033	0.4702	0.037*	
H12C	0.9508	0.3645	0.3556	0.037*	

### Atomic displacement parameters (Å^2^)

**Table:**

	*U*^11^	*U*^22^	*U*^33^	*U*^12^	*U*^13^	*U*^23^
S1	0.0269 (2)	0.0220 (2)	0.01582 (19)	0.01013 (15)	0.00368 (14)	0.00310 (13)
O1	0.0255 (5)	0.0203 (5)	0.0183 (5)	0.0113 (4)	0.0017 (4)	0.0021 (4)
O2	0.0270 (6)	0.0238 (5)	0.0175 (5)	0.0120 (4)	0.0040 (4)	0.0029 (4)
N1	0.0203 (6)	0.0153 (6)	0.0188 (6)	0.0047 (5)	0.0010 (5)	0.0011 (5)
N2	0.0205 (6)	0.0206 (6)	0.0194 (6)	0.0083 (5)	0.0000 (5)	0.0019 (5)
N3	0.0212 (6)	0.0206 (6)	0.0174 (6)	0.0088 (5)	0.0035 (5)	0.0041 (5)
N4	0.0225 (6)	0.0187 (6)	0.0141 (6)	0.0078 (5)	0.0020 (5)	0.0025 (5)
N5	0.0220 (6)	0.0190 (6)	0.0174 (6)	0.0093 (5)	0.0039 (5)	0.0006 (5)
C1	0.0230 (7)	0.0193 (7)	0.0214 (7)	0.0074 (6)	−0.0003 (6)	0.0013 (5)
C2	0.0262 (8)	0.0251 (8)	0.0235 (8)	0.0068 (6)	0.0054 (6)	0.0001 (6)
C3	0.0224 (7)	0.0236 (8)	0.0317 (8)	0.0089 (6)	0.0014 (6)	−0.0037 (6)
C4	0.0220 (7)	0.0191 (7)	0.0313 (8)	0.0081 (6)	−0.0035 (6)	0.0004 (6)
C5	0.0214 (7)	0.0183 (7)	0.0211 (7)	0.0032 (5)	−0.0020 (5)	0.0024 (5)
C6	0.0168 (6)	0.0155 (7)	0.0201 (7)	0.0034 (5)	−0.0012 (5)	−0.0016 (5)
C7	0.0172 (7)	0.0159 (6)	0.0185 (7)	0.0039 (5)	−0.0032 (5)	0.0002 (5)
C8	0.0194 (7)	0.0153 (6)	0.0171 (7)	0.0036 (5)	0.0000 (5)	−0.0004 (5)
C9	0.0186 (7)	0.0136 (6)	0.0184 (7)	0.0026 (5)	0.0005 (5)	0.0005 (5)
C10	0.0213 (7)	0.0135 (6)	0.0199 (7)	0.0042 (5)	0.0015 (5)	0.0023 (5)
C11	0.0318 (8)	0.0256 (8)	0.0194 (7)	0.0135 (6)	−0.0016 (6)	0.0021 (6)
C12	0.0249 (8)	0.0235 (8)	0.0285 (8)	0.0094 (6)	−0.0008 (6)	0.0042 (6)

### Geometric parameters (Å, °)

**Table:**

S1—C9	1.6632 (14)	C1—C6	1.394 (2)
O1—C10	1.3274 (17)	C1—H1	0.9500
O1—C11	1.4568 (17)	C2—C3	1.390 (2)
O2—C10	1.2142 (18)	C2—H2	0.9500
N1—C8	1.3194 (18)	C3—C4	1.386 (2)
N1—C7	1.3680 (18)	C3—H3	0.9500
N2—C7	1.3260 (19)	C4—C5	1.385 (2)
N2—N3	1.3674 (17)	C4—H4	0.9500
N3—C8	1.3345 (18)	C5—C6	1.397 (2)
N3—H3N	0.84 (2)	C5—H5	0.9500
N4—C9	1.3440 (18)	C6—C7	1.4685 (19)
N4—C8	1.3845 (18)	C11—C12	1.501 (2)
N4—H4N	0.84 (2)	C11—H11A	0.9900
N5—C10	1.3803 (18)	C11—H11B	0.9900
N5—C9	1.3811 (18)	C12—H12A	0.9800
N5—H5N	0.81 (2)	C12—H12B	0.9800
C1—C2	1.389 (2)	C12—H12C	0.9800
			
C10—O1—C11	115.00 (11)	C1—C6—C5	119.48 (13)
C8—N1—C7	102.43 (12)	C1—C6—C7	120.00 (13)
C7—N2—N3	102.54 (11)	C5—C6—C7	120.52 (13)
C8—N3—N2	109.22 (12)	N2—C7—N1	114.46 (13)
C8—N3—H3N	131.7 (14)	N2—C7—C6	122.90 (13)
N2—N3—H3N	118.4 (14)	N1—C7—C6	122.64 (13)
C9—N4—C8	128.91 (13)	N1—C8—N3	111.32 (12)
C9—N4—H4N	117.6 (13)	N1—C8—N4	121.13 (13)
C8—N4—H4N	113.1 (13)	N3—C8—N4	127.42 (13)
C10—N5—C9	127.10 (13)	N4—C9—N5	114.77 (12)
C10—N5—H5N	116.8 (13)	N4—C9—S1	125.65 (11)
C9—N5—H5N	115.5 (13)	N5—C9—S1	119.58 (11)
C2—C1—C6	120.04 (14)	O2—C10—O1	125.48 (13)
C2—C1—H1	120.0	O2—C10—N5	125.13 (13)
C6—C1—H1	120.0	O1—C10—N5	109.38 (12)
C1—C2—C3	120.18 (14)	O1—C11—C12	106.95 (12)
C1—C2—H2	119.9	O1—C11—H11A	110.3
C3—C2—H2	119.9	C12—C11—H11A	110.3
C4—C3—C2	119.88 (14)	O1—C11—H11B	110.3
C4—C3—H3	120.1	C12—C11—H11B	110.3
C2—C3—H3	120.1	H11A—C11—H11B	108.6
C5—C4—C3	120.28 (14)	C11—C12—H12A	109.5
C5—C4—H4	119.9	C11—C12—H12B	109.5
C3—C4—H4	119.9	H12A—C12—H12B	109.5
C4—C5—C6	120.13 (14)	C11—C12—H12C	109.5
C4—C5—H5	119.9	H12A—C12—H12C	109.5
C6—C5—H5	119.9	H12B—C12—H12C	109.5

### Hydrogen-bond geometry (Å, °)

**Table:**

*D*—H···*A*	*D*—H	H···*A*	*D*···*A*	*D*—H···*A*
N5—H5N···S1^i^	0.81 (2)	2.58 (2)	3.3739 (13)	166.0 (17)
N4—H4N···O2	0.84 (2)	1.97 (2)	2.6448 (16)	137.3 (18)
N3—H3N···S1	0.84 (2)	2.67 (2)	3.0926 (13)	113.0 (16)
N3—H3N···N2^ii^	0.84 (2)	2.32 (2)	2.9838 (18)	136.5 (18)

Symmetry codes: (i) −*x*+1, −*y*+1, −*z*; (ii) −*x*, −*y*+2, −*z*.
